# Physico-Chemical Characteristics and Microbiological Quality of Broiler Chicken Pectoralis Major Muscle Subjected to Different Storage Temperature and Duration

**DOI:** 10.3390/foods9060741

**Published:** 2020-06-04

**Authors:** Muhamad Faris Ab Aziz, Muhammad Nizam Hayat, Ubedullah Kaka, Nitty Hirawaty Kamarulzaman, Awis Qurni Sazili

**Affiliations:** 1 Department of Animal Science, Faculty of Agriculture, Universiti Putra Malaysia, Serdang 43400, Malaysia; mhd_faris@upm.edu.my (M.F.A.A.); nizamhayat88@yahoo.com.my (M.N.H.); 2 Department of Companion Animal Medicine and Surgery, Faculty of Veterinary Medicine, Universiti Putra Malaysia, Serdang 43400, Malaysia; dr_ubedkaka@upm.edu.my; 3 Department of Agribusiness, Faculty of Agriculture, Universiti Putra Malaysia, Serdang 43400, Malaysia; nitty@upm.edu.my; 4 Halal Products Research Institute, Universiti Putra Malaysia, Serdang 43400, Malaysia; 5 Institute of Tropical Agriculture and Food Security, Universiti Putra Malaysia, Serdang 43400, Malaysia

**Keywords:** broiler, storage, temperature, duration, Pectoralis major, meat quality, coliform, *Salmonella*

## Abstract

Storage temperature and duration plays an important role in meat processing. Observations in poultry processing plants have shown a serious deviation in storage condition compared to the recommended procedures. Furthermore, there is still a paucity of evidence on the effects of storage temperature and duration on meat quality and microbial population. The aim of this study was to determine the effects of different temperature and duration during storage on physico-chemical properties and microbiological quality of broiler chicken Pectoralis major muscle. Eighty birds were slaughtered and processed, following which the packed boneless breast (PBB) (each bird was to provide two breast muscle samples; left breast and right breast) was divided into four groups, each consisted of 40 PBB. Each group was subsequently assigned to storage either at 4 °C, −10 °C, −18 °C or −40 °C, for 24 h before 20 PBB samples from each group were transported to the respective laboratory for meat quality and microbiological analysis. The remaining 20 PBB from each storage temperature were stored for 72 h before being transported for analysis. Results have shown significant increases in drip loss and cooking loss as the storage temperature decreases. Similarly, storage duration significantly affected cooking loss, of which, samples stored for 72 h exhibited higher cooking loss compared to those stored for 24 h. For color, significant differences were only observed in lightness (L*) and redness (a*) values. Longer duration of storage had significantly improved tenderness whereby, samples that have been stored for a shorter duration presented higher pH values. Populations of coliform and *Salmonella* decreased significantly with decreasing temperature and increasing storage duration.

## 1. Introduction

Storage temperature is one of the most important factors throughout the processing chain mainly for meat quality preservation [1,2,3,4,5,6,7]. Carcasses that have been through a good processing practices starting from receiving, stunning, slaughtering, scalding, defeathering, chilling and packing needs to be stored at correct temperature to minimize quality loss.

Recently, there has been a renewed interest in cold storage condition. Studies about freezing temperature, freezing duration, thawing temperature and other related factors are becoming more popular due to the prevalence of more food poisoning incidence [8,9,10,11]. Existing research recognizes the critical role played by storage temperature in maintaining an optimum meat quality and safety before it reaches the consumers. Although generally, freezing does not decrease the population of microbes in meat, spoilage is still terminated because the microbes are in a dormant state. However, a combination of a good hygiene and handling practices together with proper temperature control will lead to the decline in the population of pathogenic microbe [12]. Berrang et al. [13] stated that Campylobacter counts for carcass that have been chilled after processing were lower than the one that are directly taken right after defeathering.

However, observations from previous studies have indicated a serious deviation in processing house storage temperature compared to the recommended temperature in the Hazard Analysis Critical Control Point (HACCP) manual [3,4,6,7]. The deviation has grown in importance in light of recent events related to the increase of processing cost and the final product price due to the global economic setback. Despite the importance of storage temperature, there remains a paucity of evidence on their effects on meat quality and microbial population. Leygonie et al. [2] stated that there is still an inadequate amount of information on microbial quality and shelf life for all types of meat. Although, research has been carried out on the effects of storage temperature on meat quality [1,2,3,4,5,7,10,11], inconsistencies still exist between the studies regarding the effect of freezing temperature and freezing duration and the optimum temperature and duration in order to maintain meat quality during storage, while the studies on the combination of both factors are still limited, particularly on raw poultry meat. Furthermore, most of the results were based on the assumption that the carcasses were processed according to the standards found in the Hazard Analysis Critical Control Point (HACCP) manual. In reality, storage temperature deviation from the given standard is an everyday problem. What is yet to be clarified is the impact of the deviation on the meat quality and safety in terms of microbial contamination. The current study thus, analyzed the impact of different storage temperatures on meat quality and microbial population of broiler chicken meat.

## 2. Materials and Methods

A total of 80 chickens (at 42 day) were randomly selected from a batch of 500 birds. Broilers were slaughtered and processed in a commercial poultry processing plant following standard protocols and guidelines (MS1500: 2009). Birds were slaughtered and processed in a commercial poultry processing plant following standard protocols and guidelines (MS1500: 2009). The birds were divided into four groups. At completion of each evisceration, the dressed carcasses were subjected to deboning, trimming and packing, following which the packed boneless breast (PBB); each bird provided 2 breast muscle samples (left breast and right breast). Each group consisted of 40 PBB (*n* = 160). The PBB samples were further grouped based on the cold storage treatment and placed in a basket. Each group was subsequently assigned to storage either at: (1) 4 °C, (2) −10 °C, (3) −18 °C or (4) −40 °C, for 24 h before 20 PBB samples from each group were transported to the Meat Science Laboratory, Department of Animal Science, Faculty of Agriculture, Universiti Putra Malaysia. Upon arrival, subsamples of the breast muscles were collected for instrumental meat quality analysis. The rest of the 20 PBB from each storage temperature were subjected to the same procedure but was stored for 72 h before transported to similar laboratory located in Universiti Putra Malaysia.

### 2.1. Meat Quality Analysis

#### 2.1.1. Water Holding Capacity (Drip Loss and Cooking Loss)

The meat samples were subjected to drip loss and cooking loss measurement, according to Roslan et al. [14]. For drip loss measurement, the meat samples were weighed and placed in polyethylene bags. The bags were then sealed, vacuum packed, and stored at 4 °C. After 24 h storage, the meat samples were removed from the bag and reweighed. The percentage of drip loss was determined as follows [15]:

(1) $$Drip\;loss\;\left(\%\right)=\left(\frac{\left(Wa-Wb\right)}{Wa}\right)\times 100$$

where *Wa* = Muscle weight before storage (g) and *Wb* = Muscle weight after storage (g).

For cooking loss measurement, the meat samples were weighed and placed in polyethylene bags. The bags were then submerged in an 80 °C pre-set water bath. Once the internal temperature reached 78 °C, the bags were allowed to cool by being placed under running tap water for 30 min and carefully blotted dry with paper towels without squeezing. The muscle samples were then removed from the polyethylene bags and reweighed. The percentage of cooking losses were then calculated using the following equation [15]:

(2) $$Cooking\;loss\;\left(\%\right)=\left(\frac{\left(Wx-Wy\right)}{Wx}\right)\times 100$$

where *Wx* = muscle weight before water bath cooking (g) and *Wy* = muscle weight after water bath cooking (g).

#### 2.1.2. Color

The meat color parameters (L*: lightness, a*: redness and b*: yellowness) were assessed using the ColorFlex^®^ system (Hunterlab, Reston, VA, USA) with illuminant D65 as the light source and a 10° standard observer, with 5 cm size of aperture opening according to Karami et al. [16]. Three readings were taken from each meat sample.

#### 2.1.3. Shear Force Measurement

Following each frozen storage duration (24 h and 72 h), the meat samples were thawed at 4 °C. The meat samples were then cooked in a water bath until internal temperature reached 78 °C, and further cooked for 10 min, before cooled down at 4 °C for overnight. Of each cooked sample, three replicate blocks (1 cm × 1 cm × 2 cm) were sheared as paralleled to the direction of the muscle fibers as possible, using Volodkovitch bite jaw attached to the HD plus texture analyzer (Stable Micro System, Surrey, UK).

#### 2.1.4. pH

The pH of meat samples were measured in homogenates (water in the presence of 5 mM sodium iodoacetate (Merck Schuchardt OHG, Germany)) using a portable pH meter (Mettler Toledo, AG 8603, Switzerland) and a hand held glass electrode.

### 2.2. Microbiological Analysis

The meat samples were assessed for coliform and *Salmonella* population according to the protocol by Sabow et al. [17]. On each sampling day, 5 g of chicken meat samples were aseptically weighed, transferred to a stomacher bag containing 45 mL of 2.25% of peptone water (Merk KGaA, Germany) and homogenized using a stomacher (Inter Science, France) for 120 s at room temperature. For microbial enumeration, 100 µL samples (of 10-fold dilution in peptone water were spread on the surface of dry media, on petri dishes in duplicate for enumerations of *Escherichia coli* on MacConkey agar (Merk KGgA, Germany) and *Salmonella* on xylose lysine deoxycholate agar (Merk KGgA, Germany). For all microbial counts, plates were incubated at 37 °C for 24 h. A colony was counted by a colony counter, and the data (growth counts) were transformed to log10 values.

### 2.3. Design and Statistical Analysis

All data were analyzed by two-way (ANOVA) using the Statistical Analysis System software package, version 9.3 (SAS Institute Inc., Cary, NC, USA). Mean difference was calculated using Duncan’s new multiple range test.

## 3. Results

### 3.1. Water Holding Capacity

There was a significant interaction (*p* < 0.05) between storage temperature and duration in affecting drip loss of meat samples, with higher drip loss percentages were observed on freezing temperatures after longer storage duration. Significant differences (*p* < 0.05) in drip loss were noticed among meat samples stored at different temperature, with the highest drip loss recorded following freezing at −18 °C and −40 °C, while the lowest drip loss was observed at chilled temperature (4 °C) for 24 h. The meat samples stored for 72 h did not show any significant differences in drip loss across all storage temperatures. Meanwhile, storage duration also significantly affected drip loss percentage of meat samples, of which, higher (*p* < 0.05) drip loss value was observed in the meat samples stored for 72 h compared with 24 h, only at 4 °C and −10 °C. Further reduction in temperature after both storage duration did not affect drip loss percentages in meat samples (Table 1). For cooking loss, although there was no significant interaction between the storage temperature and duration in affecting cooking loss percentages, it was demonstrated that the reduction in temperatures significantly increased (*p* < 0.05) the cooking loss percentages in meat samples subjected to both storage durations. Meanwhile, the meat samples stored for 72 h showed significantly higher (*p* < 0.05) cooking loss compared to those stored for 24 h at all temperatures, except at −40 °C (Table 2).

### 3.2. Color

There was no significant interaction between the storage temperature and duration, on the differences in lightness (L*), redness (a*) and yellowness (b*) values of the meat samples (Table 3). There was only a significant effect of storage temperature on color values, whereby, the meat samples stored at −18 °C for 24 h showed a significant (*p* < 0.05) reduction in L* compared with the samples stored at 4 °C and -10 °C. Meanwhile, the storage duration for 72 h has significantly (*p* < 0.05) reduced L* values compared with those subjected to 24 h storage duration. The meat samples stored at varying temperatures for 72 h did not show any significant differences in L* values. On the contrary with the L* values, meat samples stored at −18 °C for 24 h showed a significant (*p* < 0.05) increase in the a* values, with significant (*p* < 0.05) reduction in a* values in the meat samples stored at 4 °C at 72 h. The meat samples stored at 72 h showed significant (*p* < 0.05) reduction in a*, compared with those for 24 h storage temperature, but only for the meat samples stored at 4 °C and −18 °C. For b* values, there was only a tendency (*p* < 0.1) for the b* values to be elevated and this was observed in the meat samples stored at 4 °C at 72 h. The b* values were also significantly (*p* < 0.05) reduced after 72 h storage, and this was only noted in samples stored at −10 °C.

### 3.3. pH

No significant interaction was noted between storage temperature and duration on their effects on pH of the meat samples. Storage temperature has not affected meat pH and these were consistently seen in all storage durations. However, meat pH values were significantly affected by the storage durations (*p* < 0.05) and this was demonstrated by declined pH values after being stored for 72 h irrespective storage temperatures. The results suggest increased acidity of the meat samples over the storage duration (Table 4).

### 3.4. Tenderness (Shear Force)

In this study, there was no significant interaction between storage temperature and duration on the shear force value of the meat samples. There was also no significant effect of storage temperatures on the shear force value. Shear force value was only affected (*p* < 0.05) by storage duration, and this was noted as a significant decreases in shear force values of the meat samples over the 72 h storage duration at different temperatures (Table 5).

### 3.5. Coliform and Salmonella Population

There was a significant (*p* < 0.05) interaction between the storage temperature and duration in affecting coliform population of the meat samples (Table 6). Surprisingly, the coliform population was significantly increased (*p* < 0.05) after being stored at −18 °C for 72 h. However, no population was detected following 72 h of storage, at −40 °C. Similarly, significant interaction between storage temperature and duration was also noticed in the case of *Salmonella* population. Nonetheless, there was only a tendency (*p* < 0.1) for *Salmonella* population to be increased, after being stored for 72 h at −18 °C, but no detection of population at −40 °C (Table 7). This data showed that, reducing storage temperature until −40 °C, accompanied with extending storage duration, would have a negative impact on bacterial growth and population.

## 4. Discussion

### 4.1. Effects of Different Storage Temperature and Storage Duration on Water Holding Capacity

Water holding capacity in meat is influenced by the changes in muscle cellular and extracellular components [2]. Generally, a reduction in meat water holding capacity could be resulted from freezing, frozen storage duration and thawing [18,19]. Our study demonstrated that frozen storage temperature (−18 ℃ and −40 ℃) and longer storage duration (after 72 h storage) exhibit higher drip loss percentage, which were related to the decrease in water holding capacity. Meanwhile, similar effect was also observed in the meat cooking loss, indicating reduction in meat water holding capacity after being stored at lower temperature, and longer storage. This have been reported by previous studies suggested that the fluctuation could be due to the ice crystal formation along the freezing gradient and duration. The reduction in the storage temperature and increase in storage duration would increase the formation of the ice crystal, which ruptures the tissue membrane, and affects the water holding capacity in the muscle. This would also increase water movement from intracellular into extracellular spaces, contribute to the increases in the concentration of the solutes in the cells, leads to protein denaturation and reduces the meat ability to retain water [2,20]. Meanwhile, the presence of oxygen could also contribute to the lipid and protein oxidation, which may cause structural damage to the meat, and thus increase water loss after longer storage duration [21].

In combination, the moisture loss from cooking was higher than that from the drip loss. This was probably due to the region in the muscle tissue from which, water loss originates. Savage et al. [22] reported that the main composition of drip loss was found to be sarcoplasmic protein, and this could explain the reduction of water holding capacity, which might be due to the disruption in structure of muscle fibers by heating. However, the water loss through cooking does not differ significantly between fresh and frozen meat samples, as well as for samples frozen and thawed at different rates [1], which suggests that the water could originate from the release of chemically bound water due to fat melting and protein denaturation [18].

### 4.2. Effects of Different Storage Temperature and Storage Duration on Color

Meat color is one of the main attributes of palatability. Mancini and Hunt [23] stated that consumers indicate the meat wholesomeness and freshness through meat discoloration, which shows how important color attribute in meat purchasing decision. The current study demonstrated a significant reduction in lightness after longer storage duration (after 72 h storage), as well as in the frozen storage temperature (at −18 °C after 24 h storage), and this may be explained by aforementioned decreased meat water holding capacity, which in turn could lead to the reduction in surface light reflectivity [24,25]. In addition, the discoloration of meat is also associated with increased in oxidative processes from lipid oxidation, for the formation of metmyoglobin. The accumulation of metmyoglobin, which could be due to the reduction of metmyoglobin reducing enzymes activity relates to color deterioration, which corresponds to more discoloration and changes in light reflectivity [25,26]. Meanwhile, the changes in the enzyme activity may also affect the increased redness value at frozen storage temperature compared with chilled temperature, and this corroborates with Fernandes et al. [27], which demonstrated increase meat redness after being stored at frozen (−12 °C) compared with chilled (between 0 and 4 °C) temperature. However, the redness values following longer storage were reduced, in both chilled (4 °C) and frozen temperatures (−18 °C), suggests possible denaturation of globin over the storage duration. The meat yellowness value also tended to reduce in freezing storage temperature and significantly reduced after 72 h storage. A previous study by Augustyńska-Prejsnar et al. [25] demonstrated increased yellowness after 7 months of freezing storage of chicken breast meat, regardless the methods of thawing. However, the yellowness value also increased after 4 days of meat stored at chilled storage temperature [28]. This contradicting data, as well as limited data on yellowness values suggest that there could be an underlying mechanism, which may explain the effects of freezing storage temperature compared with chilled temperature on the meat yellowness value, which could be accompanied with storage duration and also might be related with lipid oxidation and metmyoglobin formation. Several authors had also documented negative [29] and positive [30] correlations between yellowness and pH, suggesting the underlying effects of glycolytic process and protein denaturation on the yellowness.

### 4.3. Effects of Different Storage Temperature and Storage Duration on pH

Meat pH is one of the most important meat quality parameters, which is also associated with the rate of glycolytic pathway during post slaughtering. The current study demonstrated the reduction in pH of chicken breast meat after long storage duration (after 72 h storage), in consistent with argument by Leygonie et al. [2]. After slaughtering, the anaerobic glycolysis resulted in accumulation of lactate, which contribute to the increased concentration of hydrogen ions (H^+^), as well as by the protein denaturation, which release H^+^, thus leading to the changes in muscle pH value [31,32].

The pH also affects water holding capacity, and this was already explained in Section 4.1. Denaturation of protein, which is caused by a reduction in pH, contributes to the loss of ability for the meat to hold water. This reduction in pH could be caused by the release of amino acids and carbonyls from the protein denaturation, which could have changed the myofibrillar protein isoelectrical point, thus also the loss of bounded water, and reduced water holding capacity in meat after long storage duration [1].

Meanwhile, no significant effect was recorded by the storage temperature on pH. A study by Fernandes et al. [27] also demonstrated that the pH of chilled and frozen chicken breast meat were not significantly different. Meanwhile, a study by Kandeepan and Biswas [33] demonstrated that there was a significant difference between chilled and frozen beef meat due to increased microbial activity in chilled meat. This discrepancy suggested that species could play an important role in affecting changes in the pH of the meat.

### 4.4. Effects of Different Storage Temperature and Storage Duration on Texture

Consumer acceptance may rely on meat tenderness. The current study demonstrated that meat stored for a longer storage duration (after 72 h storage), regardless of the storage temperature, has shown a significant increase in tenderness, which is in line with other studies [2,3]. Tenderness resulted mainly due to the disruption of protein structure in meat [3]. During frozen storage, the myofibrils were broken apart by the ice crystal formation, which disrupts the physical structure such reducing fiber membrane strength, resulting in tenderization [34]. Meanwhile, the protease enzymes could also probably contribute to the ice crystal formation during proteolysis, with increasing ageing [18]. It was demonstrated that the reduction in shear force value represents post-mortem tenderization after longer storage duration, which is caused by the proteolysis to compensate the energy reserves after the onset of rigor [35].

The non-significant effects of storage temperature on tenderness in the current study also are in line with a previous study by Fernandes et al. [27]. Similar results were also reported by Ji et al. [36] in pork meat. However, it has been reported that sensory evaluation of freeze/thawed beef meat was rated less tender compared with chilled meat, which might be due to the fluid loss during thawing, and reduced hydration of muscle fibers leads to a reduced tenderness [2,3]. This might suggest that the storage temperature effect on tenderness is varied, which could be affected by different species.

### 4.5. Effects of Different Storage Temperature and Storage Duration on Microbial Population

Temperature plays an important role in microbial growth, even only a minor increase in temperature could have a serious effect on meat quality [37]. As observed in this study, coliform and *Salmonella* both were detected when muscle samples were stored in 4 °C and −10 °C regardless of storage duration, indicating the meat stored at chilled temperature is less efficient in controlling microbial growths [28]. However, the current study also demonstrated that coliform and *Salmonella* growth were undetected after being stored at −40 °C after 72 h, indicating that increase storage duration of meat in frozen state would reduce the number of microbial population. The formation of the ice crystal during freezing leads to death or injury for the microbes by damaging their cell walls and membranes, thus increasing meat shelf-life [38]. Generally, cold preservation of meat is an advantage in preserving nutritional and sensory quality of meat, by reducing meat spoilage, because of the dormancy of the microbes at a lower temperature [39]. However, it also has a disadvantage, as the microbe’s activity resumes during thawing, and exposing the meat to more favorable conditions for microbial growth because thawing is a slower and less uniform process compared to freezing [2].

## 5. Conclusions

The combination of different storage temperatures and durations had significant effects on physico-chemical characteristics and microbiological quality in the Pectoralis major muscle of commercial broiler chickens, which frozen storage temperatures (−18 °C and −40 °C) and longer storage duration (72 h) increased drip loss and cooking loss, and reduced certain color parameters (lightness and redness). Meanwhile, −40 °C storage until 72 h were beneficial in inhibiting coliform and *Salmonella* population. Meanwhile, longer storage duration also demonstrated to increase the meat tenderness and reduce meat pH. This application would be recommended in order to preserve meat physico-chemical and microbiological quality, as long as it was accompanied with good handling of the processes and in hygienic condition.

## Figures and Tables

**Table 1:** Effects of storage temperatures (4 °C, −10 °C, −18 °C and −40 °C) for two different storage durations (24 and 72 h) on drip loss percentages of Pectoralis major muscle in broiler chickens.

Temperature (°C)	Storage Duration		*p*-Value (Dur)	*p*-Value (Temp × Dur)
	24 h	72 h		
	Mean	Mean		
4	1.55 ± 0.42 ^c,y^	3.97 ± 0.95 ^a,x^	<0.0001	<0.0001
−10	2.31 ± 0.66 ^b,y^	3.63 ± 0.72 ^a,x^	0.0005	
−18	3.71 ± 0.87 ^a,x^	3.41 ± 0.63 ^a,x^	0.4320	
−40	3.32 ± 0.75 ^a,x^	3.61 ± 0.69 ^a,x^	0.4057	
*p*-value (Temp)	<0.0001	0.4991		

^a–c^ means with different superscripts within the same column differ significantly (*p* > 0.05) between storage temperatures. ^x,y^ means with different superscripts within the same row are significantly different (*p* > 0.05) between storage duration. Mean is presented ± standard error of means, SEM. Temp: temperature; Dur: duration.

**Table 2:** Effects of storage temperatures (4 °C, −10 °C, −18 °C and −40 °C) for two different storage durations (24 and 72 h) on cooking loss percentages of Pectoralis major muscle in broiler chickens.

Temperature (°C)	Storage Duration		*p*-Value (Dur)	*p*-Value (Temp × Dur)
	24 h	72 h		
	Mean	Mean		
4	2.93 ± 0.65 ^d,y^	3.67 ± 0.86 ^c,x^	0.0426	0.6583
−10	4.02 ± 0.55 ^c,y^	6.07 ± 1.63 ^b,x^	0.0014	
−18	5.22 ± 1.37 ^b,y^	6.54 ± 1.23 ^b,x^	0.0357	
−40	7.61 ± 1.50 ^a,x^	8.34 ± 1.04 ^a,x^	0.2245	
*p*-value (Temp)	<0.0001	<0.0001		

^a–d^ means with different superscripts within the same column differ significantly (*p* < 0.05) between storage temperatures. ^x,y^ means with different superscripts within the same row are significantly different (*p* < 0.05) between storage duration. Mean is presented ± standard error of means, SEM. Temp: temperature; Dur: duration.

**Table 3:** Effects of storage temperatures (4 °C, −10 °C, −18 °C and −40 °C) for two different storage durations (24 and 72 h) on color characteristics (L*, a*, b*) of Pectoralis major muscle in broiler chickens.

		Storage Duration		*p*-Value (Dur)	*p*-Value (Temp × Dur)
		24 h	72 h		
Colour	Temperature (°C)	Mean	Mean		
Lightness (L*)	4	56.684 ± 1.59 ^a,x^	45.336 ± 2.29 ^a,y^	<0.0001	0.3749
	−10	56.870 ± 1.30 ^a,x^	46.282 ± 2.05 ^a,y^	<0.0001	
	−18	54.380 ± 0.42 ^b,x^	44.33 ± 0.39 ^a,y^	<0.0001	
	−40	55.618 ± 1.60 ^a,b,x^	45.612 ± 1.58 ^a,y^	<0.0001	
	*p*-value (Temp)	0.0276	0.3809		
Redness (a*)	4	2.788 ± 0.58 ^b,x^	1.832 ± 0.25 ^b,y^	0.0100	0.1971
	−10	2.334 ± 0.17 ^b,x^	2.454 ± 0.32 ^a,x^	0.4870	
	−18	3.484 ± 0.27 ^a,x^	2.494 ± 0.29 ^a,y^	0.0007	
	−40	2.478 ± 0.27 ^b,x^	2.322 ± 0.33 ^a,x^	0.4918	
	*p*-value (Temp)	0.0007	0.0117	0.0100	
Yellowness (b*)	4	13.05 ± 1.35 ^a,x^	13.014 ± 0.67 ^a,x^	0.9588	0.7493
	−10	13.116 ± 0.59 ^a,x^	11.978 ± 0.51 ^b,y^	0.0136	
	−18	12.852 ± 1.00 ^a,x^	11.894 ± 0.98 ^b,x^	0.1469	
	−40	12.488 ± 0.28 ^a,x^	12.48 ± 0.19 ^a,b,x^	0.9595	
	*p*-value (Temp)	0.6919	0.0603		

^a,b^ means with different superscripts within the same column differ significantly (*p* < 0.05) between storage temperatures. ^x,y^ means with different superscripts within the same row are significantly different (*p* < 0.05) between storage duration. Mean is presented ± standard error of means, SEM. Temp: temperature; Dur: duration.

**Table 4:** Effects of storage temperatures (4 °C, −10 °C, −18 °C and −40 °C) for two different storage durations (24 and 72 h) on pH values of Pectoralis major muscle in broiler chickens.

Temperature (°C)	Storage Duration			*p*-Value (Dur)	*p*-Value (Temp × Dur)
	0 h	24 h	72 h		
	Mean	Mean	Mean		
4	5.792 ± 0.07 ^a,x^	5.598 ± 0.08 ^a,y^	5.5 ± 0.07 ^a,y^	0.0002	
−10	5.786 ± 0.12 ^a,x^	5.65 ± 0.13 ^a,x,y^	5.566 ± 0.11 ^a,y^	0.0452	0.5125
−18	5.740 ± 0.08 ^a,x^	5.618 ± 0.11 ^a,x,y^	5.532 ± 0.1 ^a,y^	0.0175	
−40	5.822 ± 0.08 ^a,x^	5.714 ± 0.08 ^a,x,y^	5.604 ± 0.08 ^a,y^	0.0045	
*p*-value (Temp)	0.5776	0.3483	0.3560		

^a^ means with different superscripts within the same column differ significantly (*p* < 0.05) between storage temperatures. ^x,y^ means with different superscripts within the same row are significantly different (*p* < 0.05) between storage duration. Mean is presented ± standard error of means, SEM. Temp: temperature; Dur: duration.

**Table 5:** Effects of storage temperatures (4 °C, −10 °C, −18 °C and −40 °C) for two different storage durations (24 and 72 h) on shear force values (kg) of Pectoralis major muscle in broiler chickens.

Temperature (°C)	Storage Duration			*p*-Value (Dur)	*p*-Value (Temp × Dur)
	0 h	24 h	72 h		
	Mean	Mean	Mean		
4	1.754 ± 0.03 ^a,x^	1.652 ± 0.04 ^a,y^	1.48 ± 0.05 ^a,z^	0.0001	
−10	1.794 ± 0.05 ^a,x^	1.636 ± 0.03 ^a,y^	1.458 ± 0.12 ^a,z^	0.0001	0.1519
−18	1.774 ± 0.08 ^a,x^	1.628 ± 0.06 ^a,y^	1.446 ± 0.03 ^a,z^	0.0001	
−40	1.812 ± 0.04 ^a,x^	1.594 ± 0.03 ^a,y^	1.43 ± 0.03 ^a,z^	0.0001	
*p*-value (Temp)	0.7360	0.5033	0.4381		

^a^ means with different superscripts within the same column differ significantly (*p* < 0.05) between storage temperatures. ^x,y,z^ means with different superscripts within the same row are significantly different (*p* < 0.05) between storage duration. Mean is presented ± standard error of means, SEM. Temp: temperature; Dur: duration.

**Table 6:** Effects of storage temperatures (4 ℃, −10 ℃, −18 ℃ and −40 ℃) for two different storage durations (24 and 72 h) on the coliform population (log 10) of Pectoralis major muscle in broiler chickens.

Temperature (°C)	Storage Duration		*p*-Value (Dur)	*p*-Value (Temp × Dur)
	24 h	72 h		
	Mean	Mean		
4	5.073 ± 0.87 ^a,x^	5.228 ± 0.54 ^a,x^	0.7446	
−10	5.206 ± 0.36 ^a,x^	5.49 ± 0.188 ^a,x^	0.1439	0.0171
−18	4.673 ± 0.57 ^a,b,y^	5.629 ± 0.18 ^a,x^	0.0072	
−40	2.846 ± 0.17 ^b,^	nd *		
*p*-value (Temp)	0.0622			

^a,b^ means with different superscripts within the same column differ significantly (*p* < 0.05) between storage temperatures. ^x,y^ means with different superscripts within the same row are significantly different (*p* < 0.05) between storage duration. Mean is presented ± standard error of means, SEM. Temp: temperature; Dur: duration; * nd means no microbial growth detected.

**Table 7:** Effects of storage temperatures (4 ℃, −10 ℃, −18 ℃ and −40 ℃) for two different storage durations (24 and 72 h) on the *Salmonella* population (log 10) of Pectoralis major muscle in broiler chickens.

Temperature (°C)	Storage Duration		*p*-Value (Dur)	*p*-Value (Temp × Dur)
	24 h	72 h		
	Mean	Mean		
4	3.274 ± 0.47 ^a,b,x^	3.499 ± 0.14 ^a,x^	0.3425	
−10	3.718 ± 0.32 ^a,x^	3.416 ± 0.21 ^a,x^	0.1158	<0.0001
−18	2.958 ± 0.22 ^b,x^	3.305 ± 0.14 ^a,x^	0.0805	
−40	2.851 ± 0.28 ^b,^	nd *		
*p*-value (Temp)	0.0041			

^a,b^ means with different superscripts within the same column differ significantly (*p* > 0.05) between storage temperatures. ^x,y^ means with different superscripts within the same row are significantly different (*p* > 0.05) between storage duration. Mean is presented ± standard error of means, SEM. Temp: temperature; Dur: duration; * nd means no microbial growth detected.
